# Psychological Burden and Psycho-Oncological Interventions for Patients With Hepatobiliary Cancers–A Systematic Review

**DOI:** 10.3389/fpsyg.2021.662777

**Published:** 2021-05-05

**Authors:** Johanna Graf, Andreas Stengel

**Affiliations:** ^1^Department of Psychosomatic Medicine and Psychotherapy, University Hospital Tübingen, Tübingen, Germany; ^2^Section Psychooncology, Comprehensive Cancer Center Tübingen-Stuttgart, University Hospital Tübingen, Tübingen, Germany; ^3^Charité Center for Internal Medicine and Dermatology, Department for Psychosomatic Medicine, Charite - Universitätsmedizin Berlin, Corporate Member of Freie Universität Berlin, Humboldt-Universität zu Berlin and Berlin Institute of Health, Berlin, Germany

**Keywords:** anxiety, depression, psycho-oncological intervention, psychotherapy, quality of life

## Abstract

**Background:**

Worldwide, hepatobiliary cancers are frequent diseases and often accompanied by a poor prognosis. These cancers, with hepatocellular carcinoma (HCC) and cholangiocarcinoma (CHC) being the most frequent, are often associated with a considerable amount of psychological burden such as anxiety, depressiveness, and reduced health-related quality of life (HRQOL) which may lead to psychiatric comorbidities. This systematic review gives an overview on psychological burden and on the effectiveness of psycho-oncological interventions for patients with HCC and CHC.

**Methods:**

The databases PubMed, PubPsych, and PsycINFO were used and searched using the following combination of terms: (Neoplasm OR Cancer OR Tumor OR Carcinoma) AND (Psycho-Oncology OR Psychotherapy OR Psychiatr^∗^) AND (Liver OR Hepatic OR Hepatocellular OR Gallbladder OR Bile^∗^). Studies were eligible for inclusion if investigating patients affected with tumors of the liver (HCC/CHC) and using diagnostic instruments to assess mental health symptoms and research concerning specific psycho-oncological interventions. In total, 1027 studies were screened by one author with regard to title and abstracts. Afterward, the two authors of the paper discussed inclusion of possible articles.

**Results:**

Twelve studies focusing on distress, anxiety, and depression symptoms as well as quality of life among patients with HCC/CHC and three studies on psycho-oncological interventions were included. Patients suffering from hepatobiliary cancers often experience considerable psychological burden. A quarter of patients suffer from depressive symptoms; anxiety is even more common among these patients with almost 40%. The HRQOL of those affected is reduced in almost all areas, suicide rates increased and the level of distress is considerably increased in one third of patients even in comparison to those with other kinds of cancer. By psycho-oncological intervention the prevalence of depressive symptoms and anxiety can be reduced, while the quality of life and also the survival rate of patients with hepatobiliary cancer can be increased.

**Discussion and Conclusion:**

Psychological burden is high in patients with hepatobiliary cancers as reflected in high levels of depressiveness and anxiety as well as reduced quality of life. The use of psycho-oncological interventions can reduce psychological burden and increase quality of life compared to patients receiving standard support only.

**Systematic Review Registration:**

(prospero), identifier (CRD42021243192).

## Introduction

In Europe and North America cancer of the liver–with hepatocellular carcinoma (HCC) accounting for 75% and cholangiocarcinoma (CHC) for approximately 23% of all liver cancers (Mittal and El-Serag, 2013)–is a rather rare kind of cancer with an incidence of 5.1–11.6 per 100,000 population in men and 1.3–4.3 per 100,000 population in women (Kim et al., 2019; Sayiner et al., 2019). However, from a global perspective it is the fifth and ninth most common type of cancer in men and women, respectively (Ferlay et al., 2015). Especially parts of Asia and Africa with an incidence of 25–102 per 100,000 population are heavily affected (McGlynn and London, 2005; Kew, 2013). A characteristic feature of hepatobiliary cancer is the high mortality rate with an average 5-year survival rate of less than 10%, especially due to the advanced stages diseases (Llovet et al., 1999).

The risk factors for these diseases include liver cirrhosis and viral hepatitis as well as various metabolic diseases (hemochromatosis, type 2 diabetes mellitus, and obesity) and the intake of certain drugs (anabolic steroids) or the consumption of food contaminated with aflatoxin (Wild and Gong, 2010; Fan J. H. et al., 2013). Men are more frequently affected than women. The risk of disease increases with age, with the majority of patients tending to fall ill around the age of 75. Due to the geographical disparity, however, the age of onset of the disease is significantly lower in some geographical regions.

Tumors of the liver are characterized by a wide variety of rather diffuse symptoms: tiredness, loss of appetite and nausea, pain or weight loss, palpable or even visible mass in the right upper abdomen, an accumulation of ascites or an icterus accompanied by pruritus. The diagnosis with HCC, along with the subsequent long-term invasive treatments like chemotherapy, radiotherapy and/or surgery can lead to various psychological symptoms (or diseases). The burdened person can feel sad, threatened and uncertain and develop a high unmet supportive care needs (Okediji et al., 2017). After a first acute stress reaction, psychological instability can follow, which can be considered as a normal part of the psychological adaptation process (Livneh and Antonak, 2005). Both the (chronic) disease itself and the often burdensome, stressful and protracted treatment can result in a loss of autonomy, physical vulnerability and fear of death. This trajectory often results in a high level of cancer-related distress and/or clinically relevant symptoms of different mental disorders. A study compared the prevalence of anxiety disorders and depression in hospitalized patients with cancer with the prevalence found in the general population. They showed that the prevalence of both anxiety and depression disorders is approximately doubled in patients with cancer when compared with the general population (Hinz et al., 2010). A current systematic review showed that cultural background of patients with gastric cancer influenced the prevalence rate of depression. The highest prevalence rate was found in the Eastern Mediterranean followed by the Western Pacific region (Kouhestani et al., 2020).

Due to helplessness, fear and a sense of being completely overwhelmed, grief, anger and despair–not only on a somatic but also on a psychological level–the diagnosis of hepatobiliary cancer often represents a state of emergency. Until now, these burdens have been given little consideration in society and clinical routine, which can lead to missing psycho-oncological treatment. Further, no systematic review has yet addressed the psychological burden of patients with hepatobiliary cancer. Additionally, little is known about the effectiveness of psycho-oncological interventions among this patient group.

The current systematic review assesses the extent of psychological burden of patients suffering from hepatobiliary cancers (most studies are on HCC, few studies on other types of liver cancer–gallbladder cancer, intrahepatic CHC, and extrahepatic CHC–are included and labeled accordingly, definition provided in Table 1) with a major focus on depressiveness, anxiety and health-related quality of life (HRQOL) often affected under these conditions. In addition, we also describe to what extent psycho-oncological interventions are able to alleviate the psychological burden of patients with primary tumors of the liver.

**TABLE 1:** Definitions of different types of hepatobiliary cancers.

Type	Definition
Hepatocellular carcinoma	Cancer arising from the liver cells
Gallbladder cancer	Cancer arising from the gall bladder, mostly adenocarcinomas, small percent squamous cell carcinomas
Intrahepatic/extrahepatic cholangiocarcinoma	Cancer arising intrahepatic/extrahepatic bile ducts, mostly adenocarcinomas

## Materials and Methods

### Systematic Literature Search

To address the aims described above, a systematic literature search was conducted according to the PRISMA criteria (Moher et al., 2009). The databases PubMed, PubPsych, and PsycINFO were used and searched between 2 and 3 pm on March 29th 2020 using the following combination of terms: (Neoplasm OR Cancer OR Tumor OR Carcinoma) AND (Psycho-Oncology OR Psychotherapy OR Psychiatr^∗^) AND (Liver OR Hepatic OR Hepatocellular OR Gallbladder OR Bile^∗^). Further, we have searched with MESH terms, too. However, we have detected a smaller number of papers using MESH terms in our search strategy. All papers which were detected by MESH terms were also found with the combination of the mentioned terms. Our search strategies were based on keywords being in the heading or in the text.

### Eligibility Criteria

Studies were eligible for inclusion if they were published in a peer-reviewed journal, written in English and fulfilled the following modified PICOs criteria: [P] Investigation of patients affected with tumors of the liver (HCC/CHC). [I] Studies using diagnostic instruments to assess mental health symptoms and research concerning specific psycho-oncological interventions. [C] Healthy persons or patients with other tumor entities served as control groups. [O] Outcomes were influence of hepatobiliary cancer on mental symptoms (depression, anxiety, and distress) and HRQOL.

Exclusion criteria were: (1) research concerning other tumor entities with only secondary involvement of the liver, (2) studies using a highly heterogeneous population of patients with cancer without presenting data and information specific to tumors of the liver, (3) research concerning other liver diseases such as liver cirrhosis or hepatitis or other disease-related phenomena (alcoholism, transplantation medicine), and (4) individual case studies and studies looking into highly specific groups that were not representative.

### Data Collection

After the initial search duplications were eliminated. Furthermore, one author screened the papers with regard to title and abstracts. Afterward, the two authors of the paper discussed inclusion of possible articles. In a final step the studies considered relevant were read in full and, if actually relevant, were included in the study presented here. Full texts of potentially relevant studies were assessed independently regarding the PICOs criteria.

### Statistical Analyses

The included papers showed a high heterogeneity in the psychometric assessment tools and psycho-oncological interventions. Due to this, only descriptive data analyses (median point prevalence of depressiveness and anxiety across four studies and mean point prevalence of depressiveness of anxiety before and after intervention across two intervention studies) and no group comparisons were performed. SigmaStat 3.1. (Systat Software, San Jose, CA, United States) was used for these comparisons.

## Results

**FIGURE 1:**
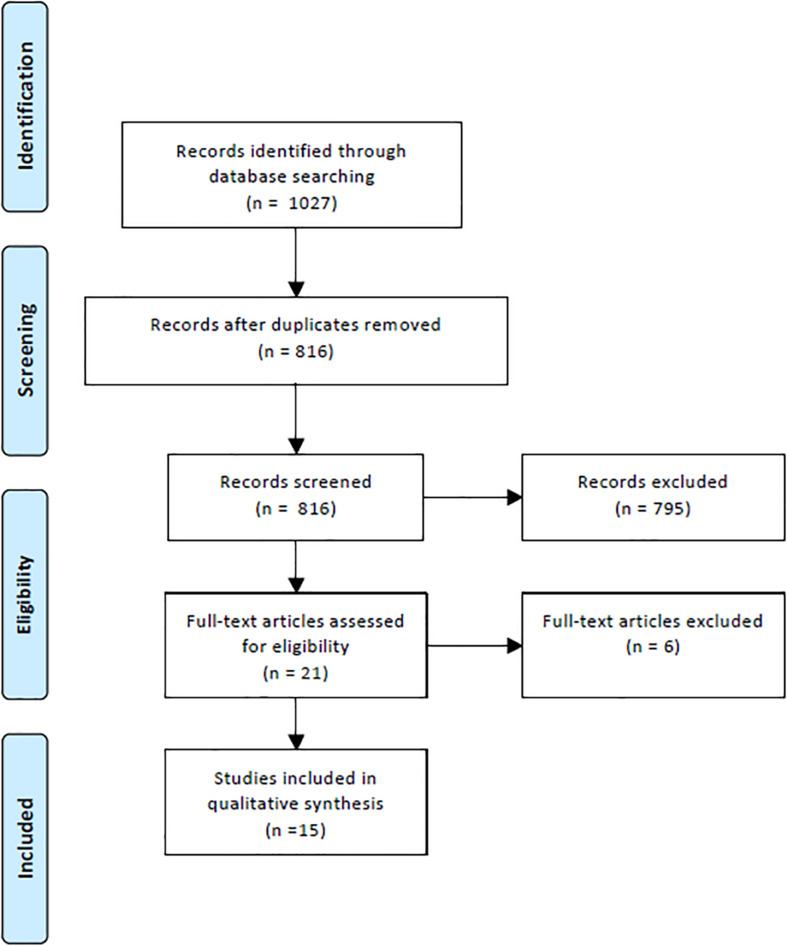
Prisma flow chart.

In the initial literature search 1027 potentially relevant papers were identified (Figure 1). After the elimination of duplicates, 816 papers remained and after reading the abstracts 795 were excluded with regard to the criteria presented above; thus, 21 papers remained. In the last step of the process, the exact reading of the full text articles, another six of the remaining studies were eliminated, leaving 15 papers that were considered eligible for the systematic review. Details on the included studies are presented in Table 2.

**TABLE 2:** List of studies included in the review.

References	Study type	Tumor entity	Number of participants	Intervention	Control	Parameters and tests	Key results	Possible bias
**Psychosocial burden**								
Zabora et al., 2001	Cross sectional	Different entities	4,496, *n* = 229 patients with liver tumors (5.1%)	None	None	-Use of the oncology center information system (OCIS): includes not only diagnosis and therapy data but also sociodemographic data etc., -Psychological distress: BSI	On average 35.1% of cancer patients with mental stress; worse prognosis and greater disease burden as risk factors, highest stress level for patients with lung, brain or pancreas tumors In patients with liver tumors 35.4% of patients affected by increased psychological stress	Response bias Selection bias
Steel et al., 2004	Call for studies	Hepatocellular carcinoma	None	None	None	None	Review of studies suggesting an immune-mediated interaction of psychosocial factors with hepatocellular carcinoma	None
Cheng et al., 2012	Longitudinal	Colon cancer or liver tumors	180, *n* = 65 with liver tumors	None	None	-Coping strategies and control conviction: CFQ, -Quality of life: SF-12 Health Survey	Significant correlation of study dropouts and low control generation/passive coping strategies; evidence that avoiding coping style and low control generation may correlate with lower quality of life.	Response bias Selection bias
Fan et al., 2010	Meta analysis, 36 studies	Hepatocellular carcinoma	798	None	None	-Quality of life: HRQOL, -Relationships between HRQOL and physical or psychological factors	In patients with HCC worse HRQOL than in chronic liver disease and normal population; strong correlation between child pugh score, liver function and HRQOL EORTC QLQ 30 + FACT-G as the most popular measuring instruments of HRQOL; Additionally 4 HCC specific questionnaires: EORTC QLQ-HCC18; Fact-Hep; FHSI, QOL-LC	Response bias Selection bias
Fielding, 2010	Call for studies	Hepatobiliary cancers	None	None	None	None	Discussion of key studies and gaps of knowledge	None
Fan S. Y. et al., 2013	Cross sectional	Hepatocellular carcinoma	286, during (inpatient) treatment	None	None	-Demographic and clinical data- -Physical condition: ECOG, -Quality of life: EORTC QLQ-C30, -Disease perception: Brief IPQ, -Coping: Jalowiec Coping Scale	Patients with HCC have reduced global HRQOL; influence of disease perception and coping strategy on HRQOL: more positive disease perception, better performance status and problem-oriented coping with positive effect on HRQOL; patients with HCC show better emotional function	Response bias Selection bias
Mikoshiba et al., 2013	Cross sectional	Hepatocellular carcinoma	128, ≥1 year after curative treatment	None	None	-Depressive symptoms: CES-D, -Quality of life: QLQ-C30 + EORTC QLQ-HCC18, -Sociodemographic and clinical data	Prevalence of depressive symptoms (CES-D 16+): 28.3%; determinants of occurrence: Karnofsky-Score, poor liver function, people living alone, unemployment; depressive symptoms correlate with significantly lower quality of life Effect sizes for the EORTC QLQ-30 scales were between 0.71 and 1.25 Effect sizes for the EORTC QLQ-HCC scales were between 0.13 and 0.90	Response bias Selection bias
Chang et al., 2015	Retrospective cohort study	Hepatocellular carcinoma	55,973	None	None	-Demographic and clinical data -Incidence of depression and exclusion of former psychiatric diagnoses	Depression incidence of 2.5%, women more affected than men; incidence significantly higher than in the general population despite a rather narrow definition of depression; risk factors for the occurrence of depression: female sex, age between 40–59 and 60–79, metastases, hepatitis C	Response bias Selection bias Recall bias
Liu et al., 2017	Longitudinal	Hepatocellular carcinoma	110, after curative liver resection	None	None	-Anxiety: HAMA/HARS, -Serum catecholamines: ELISA	56.3% with anxiety (HAMA >17); correlation with occurrence of metastases, TNM classification and hepatitis B surface antigens; correlation of catecholamine levels and HAMA score + catecholamine levels and metastases, hepatitis B, TNM classification and tumor differentiation; HAMA score and catecholamine levels associated with recurrence and poor prognosis	Response bias Selection bias
Anderson et al., 2018	Longitudinal	Gastrointestinal tumors	856,294, *n* = 81,684 hepatobiliary cancers (9.5%)	None	None	-Use of the SEER program (American Cancer Registry): collects socio-demographic and health data, including cause of death	For all types of cancer significantly increased suicidal rates compared to the normal population; especially prominent: esophageal and pancreatic cancer; for tumors of the stomach, liver and bile ducts, suicidal rates are still twice as high as in the normal population; Highest rate 2 months after cancer diagnosis	Response bias Selection bias
Jia et al., 2019	Cross sectional	Hepatocellular carcinoma	269	None	None	-Depressive symptoms: HAMD-17, -Anxiety: BAI, -Social support: SSRS, -Pain: NRS, -Quality of sleep: PSQI, -Sociodemographic and clinical data	134 patients with depressive symptoms (49.8%); relevant factors for the occurrence of depression: income, level of education, social support, anxiety score, sleep quality, pain, degree of liver cirrhosis, notification of diagnosis, AFP; development nomogram	Response bias Selection bias
Cheng et al., 2019	Meta analysis, 2 studies	Hepatocellular carcinoma	266	None	None	-Sociodemographic data, -Depressive symptoms: CES-D, -Cytokines	24% with depressive symptoms; women more frequently affected; for men, disability and unemployment as a burdening factor; gender, occupational status and income as strong predictive factors for depressive symptoms and inflammatory cytokines	None
**Psycho-oncological intervention studies**								
Kuchler et al., 2007	Randomized intervention	Gastrointestinal tumors	271, *n* = 108 hepatobiliary cancers, *n* = 45 primary liver tumors	Professional psychotherapeutic support in the sense of psycho-oncology	Routine care by employees of the surgical department	In baseline study: -Quality of life: EORTC QLQ-C30 + cancer-specific module, - survival rate	Improved survival rate and disease progression in the experimental group in stomach, pancreas, liver and colorectal cancer After 10 years follow-up: 29 patients of the experimental group and 13 of the control group survived (in patients with primary liver tumors survival ratio in 2 years 12:8, survival ratio in 10 years 9:3)	Response bias
Steel et al., 2007	Randomized intervention	Hepatobiliary cancers	28	Individually adapted psychological-psychotherapeutic care (training, behavioral therapy, supportive-expressive therapy, pharmacological intervention)	Information on disease, therapy and medication, telephone support during treatment	-Quality of life: Fact-Hep, -Depressive symptoms: CES-D, -Anxiety: STAI, -Leukocyte count, -Sociodemographic data and disease-related information -Survival rate	Clinical, but statistically not significant, effect of the intervention (minimal important difference): improvement of HRQOL, reduction of depressive symptoms and anxiety, increased leukocyte count, slightly increased life expectancy Effect sizes for the different parameters were between 0.00 and 0.20	Response bias
Wang et al., 2019	Randomized intervention	Hepatocellular carcinoma	136, after liver resection	Health training, personal conversation and support, guided patient meetings, telephone follow-up and support after the surgical therapy	Supply of patients with educational material, 60-min individualized consultation on the day of discharge, after discharge every 3 months control appointments with psychosocial focus (≥30 min)	-Anxiety + depressive symptoms: HADS, -Quality of life: QLQ-30 -Survival rate	Success of the therapy concept after 12 months: reduced number of depression and anxiety disorders (although with similar severity); improved quality of life and survival rate of the intervention group	Response bias

### Psychosocial Burden of Patients Suffering From Hepatobiliary Carcinoma

#### Depressiveness

**FIGURE 2:**
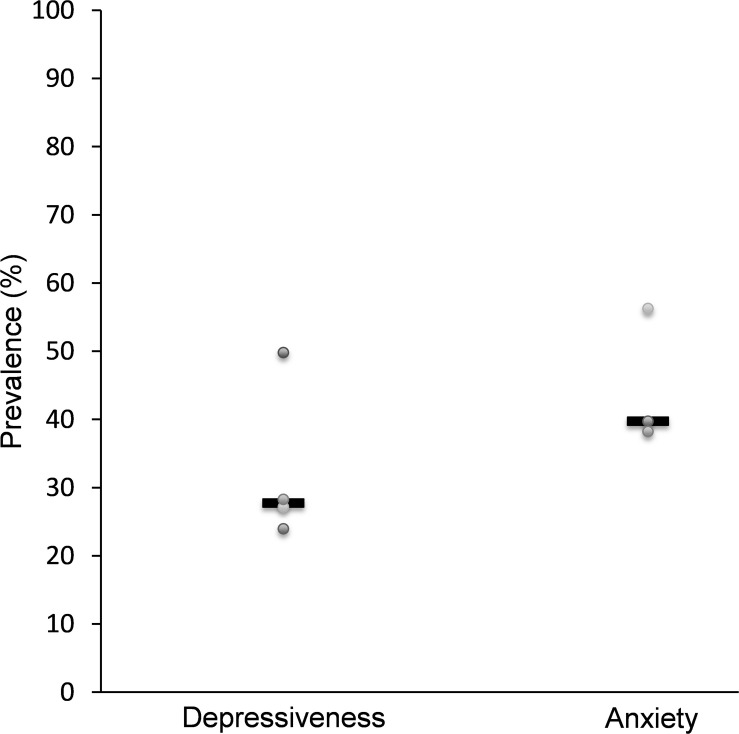
Studies assessing the prevalence of depressiveness and anxiety in patients with hepatocellular carcinoma. Data for depressiveness (*n* = 799 patients) are based on Mikoshiba et al. (2013), Cheng et al. (2019), Jia et al. (2019), and Wang et al. (2019), for anxiety (*n* = 515 patients) on Liu et al. (2017), Jia et al. (2019), and Wang et al. (2019).

Data on the prevalence of depressive symptoms were obtained from a total of four of the included studies. In two cases the psychometric tools used were the Center for Epidemiologic Studies Depression Scale (CES-D), once the Hamilton depression scale (HAMD 17) and once the Hospital Anxiety and Depression Scale (HADS). As shown in Figure 2, the average (median) prevalence of depressive symptoms in 799 patients with hepatocellular cancer examined was 27.8% (Mikoshiba et al., 2013; Cheng et al., 2019; Jia et al., 2019; Wang et al., 2019). Risk factors for the occurrence of depressive symptoms were lower level of income (Cheng et al., 2019), lower educational level, lower level of social support, low quality of sleep, more pain and higher degree of liver cirrhosis or the impairment of liver function (Jia et al., 2019).

Another study examined the incidence of depression (diagnosis according to ICD-9 or DSM-IV) in patients with HCC over a period of 6 years. After analyzing 55,973 sets of data the cumulative incidence of depression was 2.5% (Chang et al., 2015). When expressed as incidence per person-year, incidence of depressive disorders was 6.13 per 1000 person-years in patients with HCC, a value considerably higher compared to the general population in Taiwan reported to range between 1.89 and 2.58 per 1000 person-years (Chien et al., 2007). The risk factors for the occurrence of depression in this study were female sex, older age, the occurrence of metastases and hepatitis C (Chang et al., 2015).

#### Anxiety

In three of the studies identified data concerning the prevalence of anxiety among patients with hepatocellular cancer was presented (Liu et al., 2017; Jia et al., 2019; Wang et al., 2019). A total of 515 patients were examined, resulting in an average prevalence of anxiety of 39.8% (median, Figure 2). The Beck Anxiety Inventory (BAI) score, the score of the Hamilton Anxiety Rating Scale (HAMA, sometimes termed HARS) and the HADS score were used as measuring instruments. The occurrence of anxious symptoms correlated with the occurrence of metastases and tumor recurrence, the TNM classification and with the presence of hepatitis B surface antigens (Liu et al., 2017). In addition, a correlation between anxiety and serum catecholamine levels was described (Liu et al., 2017), which could give rise to a pathophysiological mechanism of the disease.

#### Health-Related Quality of Life

Data on HRQOL could be obtained from a total of five studies. In four studies the EORTC Quality of Life-C30 questionnaire was used, once supplemented by the EORTC QLQ-HCC 18 survey (Kuchler et al., 2007; Fan et al., 2010; Fan S. Y. et al., 2013; Mikoshiba et al., 2013). In the remaining study the 12-Item Short Form Health Survey (SF-12) was used (Cheng et al., 2012). All of these studies indicate that patients with liver cancer suffer from a reduced quality of life in almost all areas. The only exception is emotional functioning which was described higher in one study compared to data from healthy volunteers from different nations (Fan S. Y. et al., 2013), a finding possibly reflecting an effect of so-called post-traumatic growth (Steel et al., 2008) as part of coping with the disease. Indeed, an influence of disease perception, control conviction and coping strategies on HRQOL was described: A more positive disease perception, a high control conviction and problem-oriented coping were described to have a positive effect on HRQOL, whereas passive-avoidant coping strategies and a low control conviction had a negative effect (Cheng et al., 2012). Lastly, the reduced quality of life of patients with HCC correlated with the Child-Pugh score (used to describe and classify liver cirrhosis into different stages according to parameters of coagulation, bilirubin, albumin, ascites, and encephalopathy), the extent of restriction of the liver function and the occurrence of depressive symptoms (Mikoshiba et al., 2013). With regard to HRQOL, a meta-analysis assessed the results of 36 studies on a total of 798 patients with HCC and showed that HRQOL was significantly reduced compared to the general population (*n* = 1075) but also in comparison to HRQOL of patients with other types of cancer (*n* = 2,236) (Fan et al., 2010).

#### Suicide Rates

One of the studies selected provides data on suicide rates in patients with hepatobiliary carcinoma. After examining the data of 81,684 patients diagnosed with cancer of the liver, the suicide rate of patients affected was about twice as high (standardized mortality ratio ∼2) compared to the normal population (Anderson et al., 2018). Relevant factors for an increased risk of suicide were older age, male gender, being unmarried status and an advanced disease process with poor prognosis (Anderson et al., 2018). Additionally, an accumulation of suicides within the first 5 years after the announcement of the diagnosis has been described. Especially the first 2 months after diagnosis seem to be particularly critical (Anderson et al., 2018).

#### Psychological Distress

Another study compared psychological distress in patients with hepatobiliary carcinoma [determined using the score of Brief Symptom Inventor (BSI)] to those with other types of cancer. The burdened patients can be identified by a Global Severity Index (GSI) score ≥63 or any two subscales where the T-score is ≥63 (Derogatis and Melisaratos, 1983). According to the analysis of data of 4,496 patients with different types of cancer, lung cancer or brain tumors proved to be particularly stressful for patients (in case of lung cancer, 43.4% of patients were affected by an increased psychological distress and 42.7% of the patients with brain tumor). However, also 35.4% of patients with hepatobiliary carcinoma (*n* = 229) showed a considerable level of distress (Zabora et al., 2001).

#### Effectiveness of Psycho-Oncological Interventions

Three of the studies investigated the effects of psycho-oncological interventions in patients with gastrointestinal tumors (among those almost half with hepatobiliary cancers), hepatobiliary cancers and HCC, respectively. It is to note that these studies differed concerning the tested intervention, the control conditions and the observation period. Therefore, the respective studies will be described separately (except for effects of depressiveness and anxiety which were calculated together for two studies).

**FIGURE 3:**
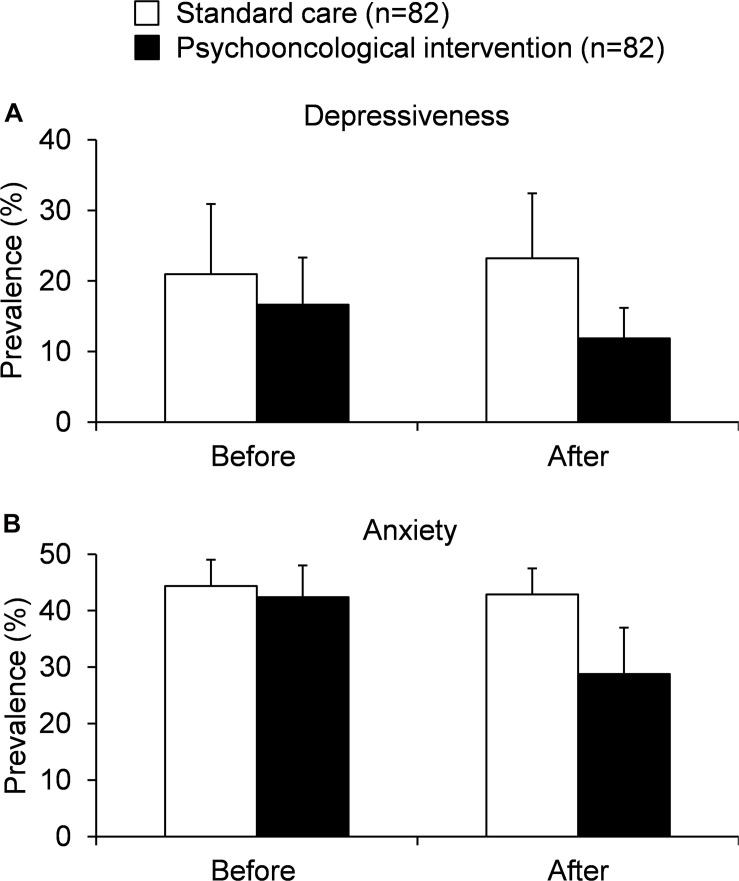
Studies assessing the effect of psycho-oncological interventions in patients with hepatobiliary cancers on depressiveness **(A)** and anxiety **(B)** [based on Steel et al. (2007) and Wang et al. (2019)].

In the first study the effect of an individually tailored psychotherapeutic care of patients with hepatobiliary cancer was tested on a total of 28 patients in a randomized longitudinal study (Figure 3). The 14 patients in the control group received routine information on the disease, therapy and medication as well as telephone support during the course of treatment, while the 14 patients in the intervention group received individually tailored psychotherapeutic care which could include training, behavioral therapy procedures (cognitive behavioral therapy, CBT), supportive-expressive therapy or closely monitored pharmacological interventions (Steel et al., 2008). The interventions involved several personal contacts and some telephone conversations between patients and medically trained personnel. The results of the 3-months follow-up survey were used to evaluate the intervention tested. Socio-demographic and disease-related data, HRQOL (Fact-Hep), the occurrence of depressive symptoms (CES-D), the occurrence of anxiety (STAI), the number of leukocyte and the survival rate were recorded. As a result of the study, the authors reported a slight improvement in HRQOL (points change from baseline vs. attention control: +17.2 vs. +11.0), a reduction in depressive symptoms (−3.2 vs. +2.0) and anxiety (−11.5 vs. −1.0, Figure 3). In addition, a slightly increased life expectancy was observed in the intervention group (Steel et al., 2008) underlining the importance of psycho-oncological support. The exact mechanisms contributing to this effect should be further determined.

In another randomized longitudinal study, the effect of a continuous bedside psychotherapeutic care of patients, which had already started pre-operatively, was tested in a total of 271 patients with gastrointestinal tumors (108 of the patients suffered from hepatobiliary cancers, 45 of them were diagnosed with a primary tumor of the liver) (Kuchler et al., 2007). The intervention included an average of six contacts with a trained psychotherapist. The main focus was on supportive therapy and crisis intervention, but topics such as rehabilitation or psychosocial aspects of the disease were also touched. In addition, patients were provided with patient-relevant information by doctors and the nursing staff. The control group received routine care by employees of the surgical department. The present study represents a 10-year follow-up in which the quality of the intervention was determined mainly in relation to the survival rate of the patients. Psychotherapeutic support resulted in an increased 2- (50.7 vs. 33.3%) and 10-year (21.3 vs. 9.6%) survival rate of the patients compared to those in the control group.

In the last randomized psycho-oncological intervention trial a total of 136 patients with HCC that underwent a hepatectomy were examined to determine the effect of psychological-educative care over a period of 1 year compared to control support (Wang et al., 2019). The intervention consisted of a combination of health training, personal interviews and support, guided patient meetings and telephone contacts following the surgical therapy. The control group on the other hand was provided with educational material, a one-time 60-min individualized consultation on the day of discharge and, following discharge, control appointments with a psychosocial focus every 3 months. The quality of the intervention was evaluated in relation to the prevalence of anxiety and depression symptoms (HADS), a change in HRQOL (EORTC QLQ-30) and survival rate. After 12 months the authors observed a reduction in the prevalence of depression (16.2 vs. 32.4%) and anxiety (20.6 vs. 38.2%) as well as an increased quality of life (points change on the health status scale: +14.4 vs. 8.0) along with an improved median overall survival of the intervention group (37 vs. 32 months) compared to patients receiving control supportive therapy (Figure 3; Wang et al., 2019).

## Discussion

Different studies show that patients with cancer suffer from severe psychological burden. However, to date, there is no clear knowledge about the impact of hepatobiliary cancers on mental health and the effect of psycho-oncological interventions in patients with hepatobiliary cancers. This is the first systematic review summarizing the psychological burden of patients with hepatobiliary cancers and the need for psycho-oncological interventions in this patient population.

From the study data presented so far two things become apparent: Firstly, patients suffering from hepatobiliary cancers often experience a considerable amount of psychological distress. About a quarter of the patients suffer from depressive symptoms (Mikoshiba et al., 2013; Cheng et al., 2019; Jia et al., 2019; Wang et al., 2019); For depression, risk factors were lower level of social status (Cheng et al., 2019), less social support, low quality of sleep, more pain and higher degree of liver cirrhosis or an impairment of liver function (Cheng et al., 2019). Furthermore, female sex, older age, the occurrence of metastases and hepatitis C were identified as risk factors (Chang et al., 2015). Anxiety is even more common among those patients with almost 40% (Liu et al., 2017; Jia et al., 2019; Wang et al., 2019). Metastases and tumor recurrence, the TNM classification and the presence of hepatitis B surface antigens significantly increased anxious symptoms (Liu et al., 2017). The HRQOL of those affected is reduced in almost all areas (Kuchler et al., 2007; Fan et al., 2010; Cheng et al., 2012; Fan S. Y. et al., 2013; Mikoshiba et al., 2013), suicide rates are increased (Anderson et al., 2018) and the level of distress is considerably increased in one third of patients even in comparison to those with other kinds of cancer (Zabora et al., 2001). The results of the review show that patients with hepatobiliary cancer suffer from high physical, psychological and social needs during the cancer trajectory. Furthermore, treatment leads to several burdensome side effects. Taken together, hepatobiliary cancer is a disease which leads to high psychological burden. With the establishment of screenings for distress and psychosocial burden, it is now possible to systematically identify burdened patients and to direct focused and resource-intensive care to those who need it most.

Secondly, the three intervention studies tentatively suggest that there might be beneficial effects of psycho-oncological interventions. The use of psycho-oncological intervention can reduce the prevalence of depressive symptoms and anxiety, while the quality of life and also the survival rate of patients with hepatobiliary cancer can be increased. The increase in survival might be due to an improvement in coping, a reduction in perceived stress and an improvement of immune functions as hypothesized by the authors (Kuchler et al., 2007). It seems that psychotherapy can improve the quality of life and this can have an influence on immunobiological processes. In addition, successful psychotherapeutic support (resulting in less psychological burden) can lead to better and more proactive health behaviors and increased compliance during follow-up care. All this can lead–directly or indirectly–to an increase in long-term survival.

These data give rise to an improvement of mental health among patients affected with HCC, but the data should be interpreted with caution. One study included only a small number of patients in the follow-up assessment (Kuchler et al., 2007). The other study initially had a small sample size and only reported clinically significant trends in the intervention group of the study (Steel et al., 2007). Moreover, it should be noted that psychological outcomes such as depression and anxiety were only assessed with self-report questionnaires in the included studies. A clinical diagnostic interview was not performed in any of the included studies. Therefore, the intervention effect on mental health is not clearly clinically objectifiable. In addition, it has to be considered that the interventions were only short-term and therefore the effectiveness should be interpreted critically. However, the results are promising and further randomized controlled studies with long-term interventions should be conducted to further investigate the effects of psycho-oncological interventions in patients with hepatobiliary cancers.

It has to be noted that any study on cancer should take into account that cancer is a complex and multifactorially determined disease. Many variables that cannot be directly controlled influence the course of the disease and the well-being of patients; therefore, it may be difficult to prove clear correlation or even causality. In addition, the patient population under investigation is also very heterogeneous: the patients differ in age, gender, socio-economic situation, their medical history, comorbidities, tumor size, number of lesions, lymph node involvement, the degree of metastasis, the therapeutic path taken and, for example, the use of alternative/complementary medical procedures. Due to the fact that equally structured interventions and control groups do not exist, a direct comparability of the effectiveness of the different studies is limited. The latter undertaking is also made more difficult by the fact that the measuring tools (questionnaires) used in the individual studies are rarely the same and even if they are, they are used differently: for example different cut-off values are used which results in different data concerning the prevalence of some symptoms. In addition, the studies also differ greatly concerning the timing of the patient interviews, meaning that patients are often interviewed at different stages of the disease. Lastly, the studies also differ in their exclusion criteria, e.g., other psychiatric diseases and related psychiatric treatment was an exclusion criterion in one study only. These factors have to be kept in mind as possible confounding variables.

Furthermore, bias cannot be ruled out for the studies included in this systematic review. We expect different risks of bias because of the different research design of the included papers. This systematic review includes studies with cross-sectional and longitudinal study designs as well as one with a retrospective study design. Only three papers reported results of a randomized controlled study design. The risk of response bias was the most frequent type of bias which occurred because all studies applied (self-reporting) questionnaires to assess psychological symptoms. Furthermore, we expect a selection bias, as it can be assumed that patients suffering from psychological symptoms are more willing to participate in this kind of studies. Lastly, recall bias might have occurred, especially in retrospective studies, because it might be that patients cannot accurately remember their symptoms during the trajectory of the disease.

Taken together, future studies should assess the effects of psycho-oncological interventions in a randomized design with desirably long follow up in well-defined/stratified patient groups and should also use–in combination with traditional face to face psychotherapeutic techniques–eHealth interventions as recently emphasized (Estape and Coups, 2020).

Even though the data presented here have to be interpreted with caution it became clear that psychosocial burden is high in patients with hepatobiliary cancers (including mostly HCC and CHC) as reflected in high levels of depressiveness and anxiety as well as reduced levels of quality of life. Psycho-oncological interventions were able to reduce perceived depressiveness and anxiety in patients with hepatobiliary cancers as well as increase quality of life compared to patients receiving standard support only. Although these data appear promising, one has to keep in mind that data especially on psycho-oncological interventions in these patients are limited so far. Therefore, future studies are warranted to further investigate the effects of psycho-oncological treatment in patients with hepatobiliary cancers (most studies will be performed in patients with HCC as this is the largest group within these cancers) to better characterize and understand the effects exerted by these procedures in order to corroborate the current recommendations of treatment guidelines already recommending psycho-oncological support (subsumed as best supportive care) for patients with hepatobiliary cancers (Benson et al., 2019).

## Data Availability Statement

The original contributions presented in the study are included in the article/supplementary material, further inquiries can be directed to the corresponding author/s.

## Author Contributions

JG performed the systematic search and wrote the first draft of the manuscript. AS planned the study and critical input throughout the study. Both authors finalized the manuscript.

## Conflict of Interest

The authors declare that the research was conducted in the absence of any commercial or financial relationships that could be construed as a potential conflict of interest.
